# Emergence of Human and Animal Melioidosis in Southern Africa, 2018–2021

**DOI:** 10.3390/tropicalmed11020060

**Published:** 2026-02-19

**Authors:** Jennifer Rossouw, Hermanus D. W. Geyer, Monica Birkhead, Douglas Wilson, Jeremy Nel, Alan S. Karstaedt, Carel E. Haumann, Annelize Jonker, Jason W. Sahl, David M. Wagner, John A. Frean

**Affiliations:** 1 Centre for Emerging Zoonotic and Parasitic Diseases, National Institute for Communicable Diseases, National Health Laboratory Service, Johannesburg 2192, South Africa; geyerhdw@ufs.ac.za (H.D.W.G.); monicab@nicd.ac.za (M.B.); johnf@nicd.ac.za (J.A.F.); 2 SA National Control Laboratory for Biological Products, Faculty of Health Sciences, University of the Free State, Bloemfontein 9300, South Africa; 3 Department of Internal Medicine, Harry Gwala Regional Hospital, University of KwaZulu-Natal, Pietermaritzburg 3201, South Africa; wilsondpk@gmail.com; 4 Division of Infectious Diseases, Department of Medicine, Faculty of Health Sciences, University of the Witwatersrand, Johannesburg 2193, South Africa; jeremy.nel@wits.ac.za (J.N.); karstaedt@mweb.co.za (A.S.K.); 5 Wits Infectious Diseases and Oncology Research Institute, Faculty of Health Sciences, University of the Witwatersrand, Johannesburg 2193, South Africa; 6 PathCare, Windhoek 10005, Namibia; 7 Department of Veterinary Tropical Diseases, Faculty of Veterinary Science, University of Pretoria, Pretoria 0002, South Africa; jonkera@ampath.co.za; 8 Ampath Veterinary Laboratories, Centurion, Pretoria 0157, South Africa; 9 Pathogen and Microbiome Institute, Northern Arizona University, Flagstaff, AZ 86011-4073, USA; jason.sahl@nau.edu (J.W.S.); dave.wagner@nau.edu (D.M.W.); 10 Wits Research Institute for Malaria, Faculty of Health Sciences, University of Witwatersrand, Johannesburg 2193, South Africa

**Keywords:** Melioidosis, *Burkholderia pseudomallei*, South Africa, Namibia, whole-genome sequencing

## Abstract

Melioidosis is increasingly recognised in tropical and subtropical regions worldwide as a serious and potentially fatal bacterial infection affecting humans and animals, acquired from the environment. Until now, human cases of melioidosis had not been reported in Southern Africa. Over a four-year period, we identified three human and two animal cases of melioidosis in South Africa and Namibia. *Burkholderia pseudomallei* isolates were investigated by matrix-assisted laser desorption/ionisation time-of-flight mass spectrometry (MALDI-TOF MS) and whole-genome sequencing (WGS). Phylogenetic analysis demonstrated substantial diversity, suggesting long-term cryptic persistence of the bacterium in the Southern African region. Limited awareness of the disease and inadequate diagnostic capacity likely contribute to its apparent rarity in the region. These findings underscore the urgent need for increased surveillance, improved diagnostics, and greater awareness of melioidosis in Southern Africa to better understand its true epidemiological burden and prevent future cases.

## 1. Introduction

Melioidosis, a serious infectious disease of humans and animals, was first described in 1912 in Rangoon, Burma (now Yangon, Myanmar) by Whitmore [1]. It is caused by *Burkholderia pseudomallei*, a Gram-negative environmental bacterium that persists as a saprophyte in soil and water. Risk factors for exposure include activities such as barefoot farming in wet conditions, outdoor agricultural or excavation work in contaminated soil, contact with floodwaters or muddy environments, and travel to regions where the organism is endemic. Many animals are susceptible to infection and can shed the organism into the environment, potentially facilitating its dissemination [2].

Underlying medical conditions such as diabetes mellitus, chronic liver or renal disease, malignancy, or immunosuppression, as well as hazardous alcohol consumption, significantly increase the risk of developing clinical disease. Human-to-human transmission is exceedingly rare. Melioidosis is often referred to as *‘the great mimicker’* because its symptoms can suggest a wide array of other diseases, making diagnosis notoriously difficult [3,4]. The spectrum of clinical presentation is protean, with infection often presenting as pneumonia, cutaneous or visceral abscesses, or fulminant septicaemia. Despite appropriate and often prolonged antibiotic therapy, case fatality rates remain high at between 10% and 50% [5]. The disease’s long and unpredictable incubation period (ranging from days to years) makes it difficult to trace the source of exposure and frequently results in misdiagnosis as tuberculosis or other acute or chronic infections. Clinical and other aspects of melioidosis have been comprehensively reviewed in the recent literature [6,7].

Melioidosis is endemic across tropical and subtropical regions, particularly northern Australia, Southeast Asia, the Western Pacific, Indian Ocean islands, and parts of South and Central America [2,8]. Across Africa, isolated human cases of melioidosis have been documented in Gabon, Cameroon, The Gambia, Kenya, Malawi, Madagascar, Eritrea, Congo, Nigeria, and Mali [2,9]. In addition, *B. pseudomallei* has recently been recovered from soil in both Nigeria and Uganda [10,11]. However, the true epidemiological burden across the African continent remains poorly defined, largely due to underdiagnosis and limited surveillance [12]. In South Africa, a single case involving a goat in 1995 remains the only previously published report [13]. Recently, local acquisition of infection in the Mississippi Gulf Coast area of the continental United States has been recognised [14], reflecting the wider geographic distribution of the pathogen than was previously assumed.

Molecular tools have enhanced the ability to track the distribution and evolution of *B. pseudomallei*. Multilocus sequence typing (MLST) has become the most widely used method to study the population structure of *B. pseudomallei* and can help determine its geographic origin [15,16]. The *B. pseudomallei* MLST scheme examines allelic variation across seven neutral housekeeping genes [17]. An alternative method based on capillary electrophoresis of the 16S–23S rDNA intergenic transcribed spacer (ITS) region has also been employed to detect geographic signals and population diversity [18,19]. However, both methods suffer limitations as high recombination rates and low resolution hamper fine-scale phylogenetic analysis [20]. Additionally, MLST can sometimes produce misleading results due to sequence type (ST) homoplasy, as unrelated strains may appear genetically similar [21]. Advancements in whole-genome sequencing (WGS) have transformed microbial epidemiology, offering improved resolution for population structure analysis. WGS enables the extraction of MLST and ITS profiles, while also allowing detailed single-nucleotide polymorphism (SNP)-based phylogenies. A combined approach incorporating MLST, ITS, and WGS offers greater accuracy in mapping evolutionary relationships and addressing the limitations posed by individual genotyping methods [20].

In this study, we describe five cases of melioidosis diagnosed in humans and animals across Southern Africa between 2018 and 2021. By presenting clinical, epidemiological, and molecular data, we aim to explore the potential endemicity of melioidosis in this under-recognised region and contribute to understanding its broader geographic and genetic diversity. These findings challenge current assumptions about the pathogen’s geographic distribution and highlight the need for increased awareness and targeted surveillance in Southern Africa.

## 2. Materials and Methods

**Figure 1:**
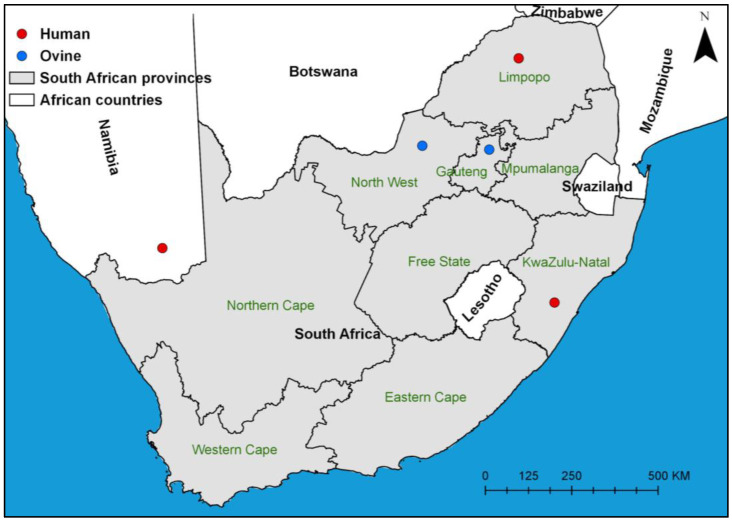
Map showing the origin of *Burkholderia pseudomallei* isolates included in this study. Coloured markers indicate human (red) and ovine (blue) cases from South Africa and Namibia (created with ArcGIS Pro 2.3.0).

Three human cases of melioidosis were identified in South Africa (*n* = 2) and Namibia (*n* = 1), and two ovine cases were diagnosed in South Africa during the study period (Figure 1).

### 2.1. Human Cases

*Case-patient 1.* A 36-year-old male construction worker in KwaZulu-Natal Province was admitted to a Pietermaritzburg hospital after presenting with dizziness, nausea, weakness, coughing, and inability to walk. The patient had previously been diagnosed with HIV infection and had interrupted antiretroviral therapy. His CD4 T-lymphocyte count was 18 cells/mm^3^ at the time of admission. He was clinically diagnosed with pneumonia and treated with amoxicillin–clavulanic acid and azithromycin. He also had perioral vesicular dermatitis that progressed to abscesses. Response to treatment was poor, and, despite sputum testing negative on the GeneXpert system (Cepheid, Sunnyvale, CA, USA), the patient was started on tuberculosis treatment empirically. His condition continued to deteriorate, and treatment with piperacillin–tazobactam was commenced. The patient died 11 days after admission. *Burkholderia pseudomallei* (isolate S-547) was subsequently isolated from an aerobic blood culture and identified on the VITEK^®^ 2 (bioMérieux, Marcy-l’Étoile, France) automated microbiology system. 

**Figure 2:**
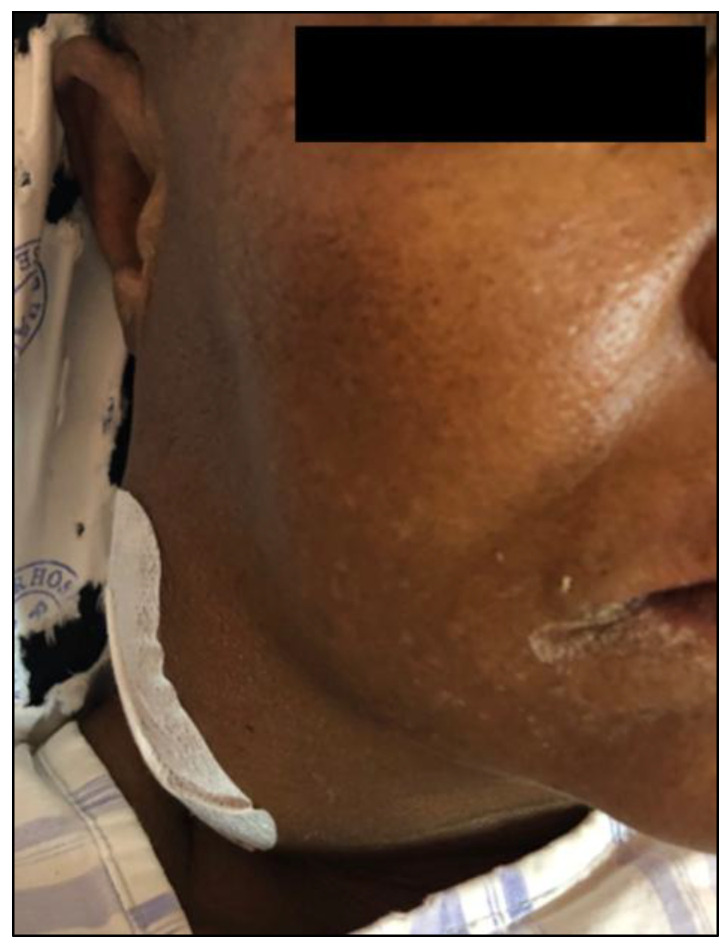
Clinical presentation of melioidosis in case-patient 2, showing a large, fluctuant swelling on the right lateral aspect of the neck, indicative of an abscess.

*Case-patient 2.* A 63-year-old female from Bochum, Limpopo Province, was admitted to hospital with a large (11 cm × 8 cm) fluctuant mass on the right lateral aspect of her neck (Figure 2). The patient experienced only intermittent pain and had no fever or other pulmonary, gastrointestinal or neurological symptoms. She had poorly controlled type 2 diabetes (HbA1c: 10.0%), chronic hypertension, and chronic kidney disease (baseline estimated glomerular filtration rate: 34 mL/min/1.73 m^2^, and urine protein/creatinine ratio: 0.342). She was initially treated with imipenem for a carbapenemase-producing *Klebsiella pneumoniae* urinary tract infection. An aerobic blood culture grew *B. pseudomallei* (isolate S-553), identified on the VITEK^®^ 2 automated microbiology system. Susceptibility testing with the VITEK^®^ 2 automated microbiology system revealed resistance to trimethoprim–sulfamethoxazole, gentamicin and tobramycin, with susceptibility to ciprofloxacin, ceftazidime, cefepime, amikacin, and piperacillin–tazobactam, as interpreted according to CLSI criteria. The abscess was aspirated, and treatment with meropenem was initiated. The patient was discharged well. She did not attend her scheduled follow-up appointment.

**Figure 3:**
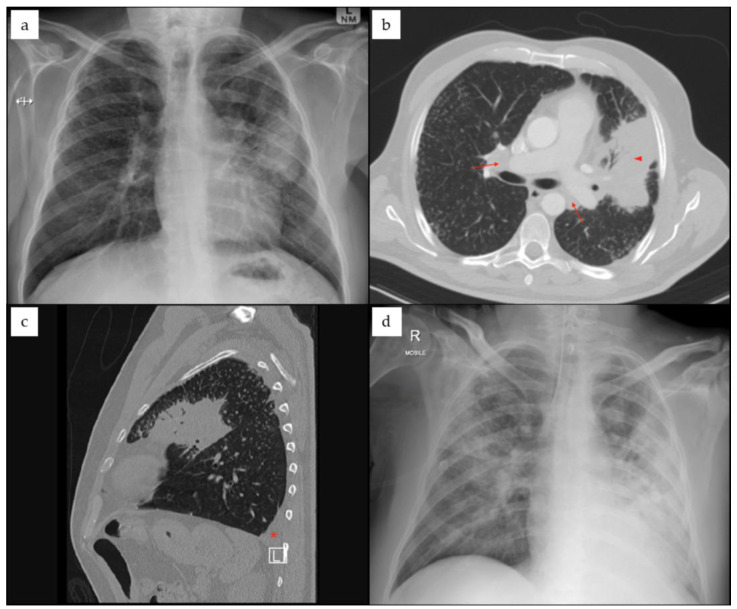
Case patient 3, chest radiology: (**a**) chest X-ray at first presentation showing left upper lobe consolidation and hilar lymphadenopathy. (**b**,**c**): CT transverse and sagittal plane images, showing left upper lobe consolidation (arrowhead), hilar lymphadenopathy (arrows) and small basal pleural effusion (*); nodular changes are present in both lungs. (**d**) Chest X-ray eight weeks later on admission, showing more extensive pneumonic infiltrates bilaterally, more on the left side.

*Case-patient 3.* A 52-year-old male farmer from the ǁKharas Region in southern Namibia was admitted to hospital after presenting with loss of consciousness, respiratory distress and renal failure. He was a non-smoker with a history of type 2 diabetes (HbA1c: 7.9–9.4%). Six weeks prior, he had visited a physician for investigation of possible pulmonary tuberculosis or malignancy. Chest X-ray showed left upper lobe consolidation and lymphadenopathy (Figure 3). He was treated with corticosteroids for a suspected non-resolving atypical pneumonia. One week prior to hospital admission, he developed a fever and worsening cough. Upon hospitalisation, he was intubated and treated with amoxicillin–clavulanic acid and azithromycin empirically for three days, after which he was switched empirically to meropenem and linezolid. *Burkholderia pseudomallei* (isolate S-563) was isolated from blood cultures and a tracheal aspirate, and was identified on the VITEK^®^ Compact (bioMérieux, Marcy-l’Étoile, France). Minimum inhibitory concentration (MIC) testing demonstrated susceptibility to meropenem (interpreted using EUCAST criteria), imipenem, ceftazidime, and trimethoprim–sulfamethoxazole (interpreted using CLSI criteria). Although the patient initially recovered and was discharged, follow-up revealed that he subsequently died four months after discharge. The cause of death was unknown.

### 2.2. Animal Cases

*Ovine case 1.* A 5-year-old female sheep (mutton merino) from a smallholding in Pretoria, Gauteng Province, presented with pneumonia that was not improving despite empiric antibiotic treatment. *Burkholderia pseudomallei* (isolate B1290-18) was isolated from a respiratory sample in mixed culture with *Corynebacterium pseudotuberculosis* and preliminarily identified by API 20 NE (bioMérieux, Marcy-l’Étoile, France) by a university veterinary diagnostic laboratory.

*Ovine case 2.* A lamb (unknown breed) from a farm in the Groot Marico district, North West Province, presented with diffuse acute necropurulent bronchopneumonia. In addition, the lamb had acute necrotic enteritis that was found to be associated with coccidiosis. *Burkholderia pseudomallei* (isolate BC1017) was isolated from a lung lesion and preliminarily identified by API 20 NE by a university veterinary diagnostic laboratory. 

### 2.3. Confirmatory Testing

The five *B. pseudomallei* isolates for the human and animal cases (Table 1) were referred to a high biocontainment facility at the National Institute for Communicable Diseases for definitive identification and characterisation by matrix-assisted laser desorption/ionisation time-of-flight mass spectrometry (MALDI-TOF MS) and whole-genome sequencing (WGS). The isolates were sub-cultured onto 5% blood agar and incubated at 35–37 °C for 24–48 h, followed by solvent inactivation [22] prior to analysis using the Bruker MALDI Biotyper (MBT; Bruker Daltonics GmbH, Bremen, Germany), with reference to the MBT Compass database (DB7854) and the security-relevant library (BTyp2.0Sec.Library 1.0). Because MALDI-TOF MS could not reliably distinguish *B. pseudomallei* from the closely related *B. mallei*, the isolate from case-patient 1 was further examined by transmission electron microscopy to assess the presence of lophotrichous flagella and general ultrastructure [23].

### 2.4. Whole-Genome Sequencing

For whole-genome sequencing, the genomic DNA was extracted from the culture isolates using the QIAamp DNA Mini kit (Qiagen, Hilden, Germany) using the protocol for isolation of genomic DNA from Gram-positive bacteria according to the manufacturer’s instructions. DNA libraries for four of the isolates (S-547, S-553, B1290-18 and S-563) were prepared using a Nextera XT DNA library preparation kit (Illumina, San Diego, CA, USA), followed by 2 × 300 paired-end sequencing runs with 150× coverage on the Illumina MiSeq or NextSeq 1000/2000 systems (Illumina, San Diego, CA, USA). One DNA sample (BC1017) was sent to a commercial sequencing facility (Inqaba Biotechnical Industries (Pty) Ltd., Pretoria, South Africa) for sequencing on the PacBio Sequel IIe system (Pacific Biosciences of California, Inc., Menlo Park, CA, USA). De novo assembly of the Illumina data was performed with SPADES v3.11.1 (S-547, S-553) or SPAdes v3.15.5 (B1290-18, S-563, S-569) [24]. Assembly of the PacBio data (BC1017) was performed with SMRTLINK v10.1.

Genome sequences were submitted to the *B. pseudomallei* MLST database (https://pubmlst.org/bpseudomallei/) (accessed on 29 January 2026) to assign the new allele and sequence types. All sequences were deposited in GenBank (accession numbers: SRRR00000000 (S-547), WEPU00000000 (S-553), JBSVKJ000000000 (S-563), JBSVKH000000000 (B1290-18), and JBSVKK000000000 (BC1017)).

### 2.5. Genomic Analysis

#### 2.5.1. Core-Genome SNP Phylogenetic Analysis

A total of 103 *B. pseudomallei* genome assemblies were aligned against the reference genome (K96243; GCA_000011545.1) with minimap2 v2.29 [25], and SNPs were called with GATK v4.5.0.0 [26]. Any SNP falling within a duplicated region in the reference, based on a NUCmer [27] self-alignment, was filtered from downstream analyses. All of these methods were wrapped by NASP v1.2.1 [28]. A maximum-likelihood phylogeny was inferred from a concatenation of 72,854 core-genome SNPs with IQ-TREE v2.3.6 [29] using the TVM + F + ASC + R9 substitution model [30].

#### 2.5.2. Molecular Typing

For each genome, in silico multi-locus sequence typing was performed by extracting BLAST v2.7.1+ [31] alignments against the pubMLST database [17]. If a given allele was novel or the allele combination had not been previously seen, then the genome was considered ‘STnovel’. If the full length of a given allele was not identified through BLASTn, then the genome was considered as ‘STtruncated’.

For ITS typing and screening of the *Yersinia*-like fimbrial (YLF) and *Burkholderia thailandensis*-like flagellum and chemotaxis (BTFC) gene clusters, reference ITS [19] and YLF/BTFC sequences [32] were aligned against all genomes with blat v35x1 [33] in conjunction with LS-BSR v1.2.0 [34]. For each genome, the variant with the largest BLAST score ratio [35] value was applied. If a BSR value < 0.9 was calculated across all ITS variants, then an ‘unknown’ label was applied.

## 3. Results 

### 3.1. Confirmatory Testing

**Figure 4:**
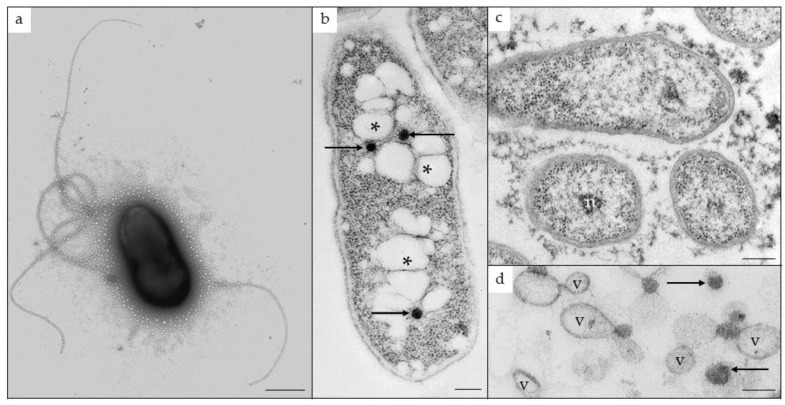
Transmission electron microscopy of *Burkholderia pseudomallei* cultured isolate. (**a**) Negatively stained, lophotrichous cell. (**b**) Sectioned cells contained polyhydroxybutyrate inclusions (asterisks) and bacterial microcompartments/metabolosomes (arrows). (**c**) A delicate filigree of exopolysaccharides surrounds cells stained with a cationic dye (n = part of a nucleoid). (**d**) Production of extracellular vesicles (v) is another known virulence factor. Vesicle clusters appeared together with metabolosomes (arrows). Scale bars = 250 nm (**a**–**c**); 100 nm (**d**).

All five clinical isolates were identified by MALDI-TOF MS as *B. mallei/B. pseudomallei*. These two closely related species could not be reliably distinguished using this system. However, unlike *B. mallei*, *B. pseudomallei* is motile and possesses lophotrichous flagella. The isolate from case-patient 1 (S-547) was referred for transmission electron microscopy, and on negative staining revealed flagellated cells (Figure 4a). Resin-embedded, sectioned cells illustrated the presence of polyhydroxybutyrate granules, as well as the production of exopolysaccharides and extracellular vesicles, both of which are known virulence factors (Figure 4b–d) [36]. 

### 3.2. Genomic Analysis

#### 3.2.1. Core-Genome SNP Phylogenetic Analysis

**Figure 5:**
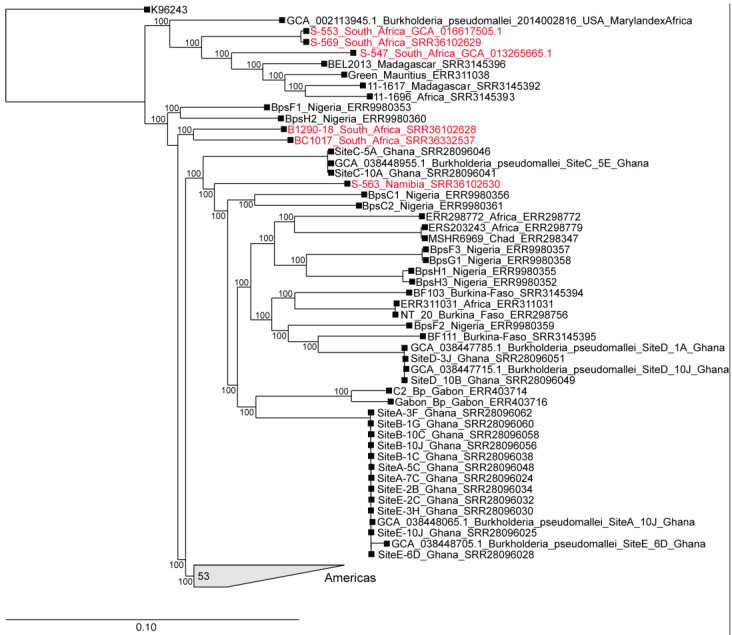
A maximum-likelihood phylogeny of core-genome SNPs from genomes in the Africa/America clade. Genomes shown in red were sequenced in the current study.

Core-genome phylogenetic analysis revealed that the Southern African *B. pseudomallei* isolates from this study clustered into two distinct major subclades within the broader African clade (Figure 5 and Figure S1). The South African human isolates (S-547, S-553) grouped within a subclade alongside East African isolates from Madagascar and Mauritius. Isolate S-553 was very closely related to another South African human isolate (S-569) that was sent to NICD for identification in 2025. However, no provenance information for the latter isolate was available. The Namibian human isolate (S-563) clustered with West African strains from Nigeria, Burkina Faso, and Ghana, and with Central African strains from Gabon and Chad in a separate subclade. Although the ovine isolates also fell within the African clade, they were phylogenetically distinct from the other strains. These ovine isolates formed sister taxa, yet remained notably divergent from each other.

#### 3.2.2. Molecular Typing

Molecular analysis, including MLST, ITS typing, and detection of the YLF gene cluster, was derived directly from WGS data. MLST showed variation across all loci, with notable diversity in *gltB* and *gmhD*, where multiple alleles were present (Table 2). The two human isolates from South Africa (S-547, S-533) belonged to STs 1720 and 1730, while the two animal isolates (B1290-18, BC1017) were assigned to STs 1803 and 92. STs 1730, 1803, and 92 were triple-locus variants of ST 1720. Additionally, ST 1803 was a double-locus variant of ST 92, and STs 1803 and 92 were quadruple-locus variants of ST 1730. The human isolate from Namibia (S-563) belonged to ST 2315 and was notably distinct, showing variation across multiple loci compared to the other four isolates. ST 2315 differed at three or more loci, highlighting its genetic divergence. Four of the five STs were unique, with no known matches in the *B. pseudomallei* MLST. This includes previously characterised strains from Africa. Additionally, ST 2315 possesses a novel allele for *gmhD*. The ITS typing showed that four of the isolates were ITS type E sequences, while one human isolate was ITS type C. The YLF gene cluster, predominantly found among isolates of Southeast Asian origin, was present in all five isolates [37].

## 4. Discussion

The predicted environmental suitability for *B. pseudomallei* in South Africa and Namibia is low, and is more favourable in higher-rainfall parts of the neighbouring countries of Botswana and Mozambique [38]. However, South Africa has been classified as a potentially endemic country, where diagnostic and reporting capacity should be strengthened [38]. Despite this classification, diagnostic capacity for *B. pseudomallei* in South Africa remains limited. No specific testing for *B. pseudomallei* is currently available, and diagnosis relies on routine blood culture methods supplemented by automated identification systems. Problems with inaccurate species identification using certain automated commercial biochemical identification systems have been well documented. These limitations have led to misidentification of *B. pseudomallei* as *Pseudomonas* species or other members of the *Burkholderia* genus [39,40]. Clinicians and laboratory scientists should remain vigilant, as misidentification of *B. pseudomallei* as *B. cepacia* continues to occur with the Vitek 2 automated biochemical identification system [39,40,41]. This issue has been highlighted in regional studies, underscoring the need for confirmatory diagnostic approaches [41]. The lack of targeted diagnostic capability, coupled with low laboratory and clinical awareness, likely contributes to the under-recognition and under-reporting of melioidosis. These limitations impede timely clinical management, hinder surveillance efforts, and constrain public health response. In addition, socioeconomic disparities and limited access to healthcare services in rural and underserved areas may further exacerbate diagnostic delays and underdetection of cases.

These diagnostic gaps are particularly concerning given the occurrence of locally acquired human and animal cases in South Africa and Namibia. None of the human or animal cases described here had originated or travelled outside South Africa or Namibia. All the human cases had underlying chronic conditions that potentially predispose to melioidosis, as well as having plausible environmental exposure. An important limitation of this study is the lack of environmental sampling from case locations, which restricts our ability to confirm local reservoirs of *B. pseudomallei*. However, the prolonged incubation periods (days to years) of melioidosis can make it difficult to identify the likely source of infection. Case-patient 1 was a construction worker with possible occupational exposure to contaminated soil or water, and had HIV-related immunodeficiency. While HIV does not appear to be a major risk factor for melioidosis, cases of such co-infection have been reported, sometimes involving multiple pathogens [42,43]. Case-patient 2, who lived in a small rural village, reported that she routinely washed clothes in a nearby river. As a farmer, she often worked barefoot in the fields. She also described heavy rainfall in the period leading up to her illness, which may have contributed to environmental exposure risks. North of the Tropic of Capricorn (23.4°S), this village is in the same general area where a case of caprine melioidosis was previously reported [13]. Case-patient 3 died four months after discharge from an unknown cause. Although definitive attribution was not possible, relapse or a delayed complication of melioidosis cannot be excluded, as late presentations and recurrent disease are well-recognised [44]. Exposure to livestock in a farm environment could account for the source of infection for case-patient 3. The patient also reported recent flooding on this farm. The ovine cases were unrelated and from two widely separated areas, neither of which corresponded with the earlier caprine case mentioned above. The two animal cases had concurrent infections with other bacterial or parasitic diseases that can weaken immunity and predispose them to secondary infections. Unfortunately, no exposure history was available for the sheep.

Recent studies have revealed considerable genetic variability and support the endemicity of melioidosis in Africa [11,20,45]. Similarly, phylogenetic analysis of *B. pseudomallei* isolates from this study demonstrated substantial diversity, indicating the long-term persistence of the bacterium in the Southern African region. These Southern African *B. pseudomallei* isolates belong to the African clade previously reported [20]. The human isolates clustered into two major subclades: one comprising the South African and East African isolates, and the other comprising the Namibian, together with West and Central African isolates. Although the ovine isolates also fell within the African clade, they were phylogenetically distinct from the other isolates. However, the small number of isolates analysed limits the strength of any broad evolutionary conclusions that can be drawn. Taken together, these findings reflect not only the endemicity and long-term establishment of *B. pseudomallei* but also its complex evolutionary history across the African continent.

MLST analysis also revealed substantial genetic diversity among the five Southern African *B. pseudomallei* isolates, with allelic variation observed across all loci. The Namibian human isolate (ST 2315) was markedly divergent, differing at three or more loci from the South African sequence types. The absence of matches for four of the five STs in the *B. pseudomallei* MLST database, including among previously characterised African isolates, highlights the underrepresentation of Southern African isolates in global genomic repositories and suggests the existence of region-specific diversity. Surprisingly, the dominant ITS type among our strains was type E. This contrasts with previously reported ITS types for African *B. pseudomallei*, which include types G, C, and CE from Madagascar; types G and C from Kenya; type GC from Mauritius; and types G and C from Burkina Faso [20]. ITS type E clusters phylogenetically with Southeast Asian strains, and to our knowledge, this is the first report of ITS type E in *B. pseudomallei* from Africa. In addition, all isolates harboured the YLF gene cluster, consistent with findings that YLF is predominantly associated with *B. pseudomallei* strains from regions outside Australia [18]. The YLF cluster is typically found in Southeast Asian strains, whereas the BTFC gene cluster is more commonly detected in Australian strains [37].

The global burden of melioidosis, expressed as disability-adjusted life years, was estimated in 2015 as 4.6 million, exceeding that of many other tropical diseases [45], and there has been a call for this infection to be recognised as a neglected tropical disease to highlight the need to reduce its global burden [46]. The factors responsible for the apparent increasing global endemicity of melioidosis have been succinctly reviewed [2,7]. In summary, epidemiological transitions, namely changing patterns of life expectancy and causes of disease, are leading to increasing prevalence of comorbidities like diabetes, chronic renal disease and alcoholic liver disease. This expands the number of people at risk, particularly in low- and middle-income countries. Zoonotic melioidosis can be spread by importation of exotic animals and livestock from endemic areas; some commonly traded livestock animals (goats, sheep, camelids) are susceptible. Climate changes manifesting as extreme weather events like typhoons followed by flooding have resulted in clusters of cases, and these events may also activate the pathogen, where it survives latently in the environment. Global human and animal migration patterns will be altered by climate change; likewise, changing agricultural practices may likely increase soil suitability for *B. pseudomallei* [2]. Future studies incorporating systematic environmental sampling and region-specific climate analyses will be critical to strengthening evidence of endemicity and are essential for understanding the ecological drivers of *B. pseudomallei* emergence in Southern Africa.

The global burden of melioidosis, expressed as disability-adjusted life years, was estimated in 2015 as 4.6 million, exceeding that of many other tropical diseases [45], and there has been a call for this infection to be recognised as a neglected tropical disease to highlight the need to reduce its global burden [46]. The factors responsible for the apparent increasing global endemicity of melioidosis have been succinctly reviewed [2,7]. In summary, epidemiological transitions, namely changing patterns of life expectancy and causes of disease, are leading to increasing prevalence of comorbidities like diabetes, chronic renal disease and alcoholic liver disease. This expands the number of people at risk, particularly in low- and middle-income countries. Zoonotic melioidosis can be spread by importation of exotic animals and livestock from endemic areas; some commonly traded livestock animals (goats, sheep, camelids) are susceptible. Climate changes manifesting as extreme weather events like typhoons followed by flooding have resulted in clusters of cases, and these events may also activate the pathogen, where it survives latently in the environment. Global human and animal migration patterns will be altered by climate change; likewise, changing agricultural practices may likely increase soil suitability for *B. pseudomallei* [2]. Future studies incorporating systematic environmental sampling and region-specific climate analyses will be critical to strengthening evidence of endemicity and are essential for understanding the ecological drivers of *B. pseudomallei* emergence in Southern Africa.

## 5. Conclusions

We report unprecedented human and animal cases of melioidosis in South Africa. These cases suggest that improved local awareness of melioidosis amongst human and animal healthcare professionals is needed, as well as better ability of routine pathology laboratories to identify possible *B. pseudomallei*, and, if necessary, refer such isolates for further identification and characterisation. Substantial genetic diversity was identified among the Southern African strains, suggesting long-term *B. pseudomallei* but cryptic endemicity in this region. It is critically important that melioidosis is recognised due to the intrinsically antimicrobial-resistant nature of the bacterium and the potentially high mortality rate of infection, especially in immunocompromised individuals and/or when diagnosis is delayed, or treatment is inadequate. South African veterinary regulations require reporting of animal melioidosis, but at present, human infection is not a statutory notifiable medical condition. If human infections continue to emerge locally, then melioidosis may need to be added to the list of notifiable medical conditions.

## Figures and Tables

**Table 1:** Summary of clinical and veterinary isolates of *Burkholderia pseudomallei* in Southern Africa (2018–2021).

Isolate Number	Year	Location	Source	Predisposing Conditions	Disease
S-547	2018	KwaZulu–Natal Province, South Africa	Human	HIV	Pneumonia
S-553	2019	Limpopo Province, South Africa	Human	Type 2 diabetes Chronic kidney disease	Soft tissue abscess
S-563	2021	‖Kharas Region, Namibia	Human	Type 2 diabetes	Pneumonia
B1290-18	2018	Gauteng Province, South Africa	Ovine	Concurrent infection (*C. pseudotuberculosis*)	Pneumonia
BC1017	2021	North West Province, South Africa	Ovine	Concurrent infection (Coccidiosis)	Bronchopneumonia

**Table 2:** Molecular analysis of *Burkholderia pseudomallei* isolates from Southern Africa, 2018–2021.

Isolate No.	Allelic Profiles							Sequence Type	ITS Type (bp)
	*ace*	*gltB*	*gmhD*	*lepA*	*lipA*	*narK*	*ndh*		
S-547	1	1	3	2	5	2	1	1720 ^1^	E (662 bp)
S-553	4	1	3	2	1	1	1	1730 ^1^	C (622 bp)
S-563	1	18	152 ^2^	1	5	1	3	2315 ^1^	E (661 bp)
B1290-18	1	3	2	2	5	1	1	1803 ^1^	E (661 bp)
BC1017	1	1	2	1	5	1	1	92	E (662 bp)

^1^ Novel sequence type. ^2^ Novel allele.

## Data Availability

The whole-genome sequence data generated in this study have been deposited in the NCBI BioProject database. The datasets are accessible under BioProject IDs PRJNA531080 (S-547), PRJNA577037 (S-553), and PRJNA1366566 (B1290-18, BC1017, S-563, S-569).

## Supplementary Materials

The following supporting information can be downloaded at: https://www.mdpi.com/article/10.3390/tropicalmed11020060/s1, Figure S1: maximum-likelihood phylogeny of core-genome SNPs from genomes within the Africa/America clade. Genomes sequenced in the present study are highlighted in red. While the American clade is collapsed in the main text for clarity, here, the expanded topology is shown to display the detailed relationships among genomes from the Americas.

## Abbreviations

The following abbreviations are used in this manuscript:

BTFC	*Burkholderia thailandensis*-like flagellum and chemotaxis
ITS	Intergenic transcribed spacer
MALDI-TOF MS	Matrix-assisted laser desorption/ionisation time-of-flight mass spectrometry
MLST	Multi-locus sequence typing
ST	Sequence type
SNP	Single-nucleotide polymorphism
WGS	Whole-genome sequencing
YLF	*Yersinia*-like fimbrial
