# Supplementary material for: Emergence of Human and Animal Melioidosis in Southern Africa, 2018–2021
Source: Trop Med Infect Dis. 2026 Feb 19;11(2):60. doi: 10.3390/tropicalmed11020060 (PMC12945296; doi:10.3390/tropicalmed11020060)
Supplement: Supplementary file 1 [file tropicalmed-11-00060-s001.zip › tropicalmed-4128325-supplementary.pdf]

Supplementary material

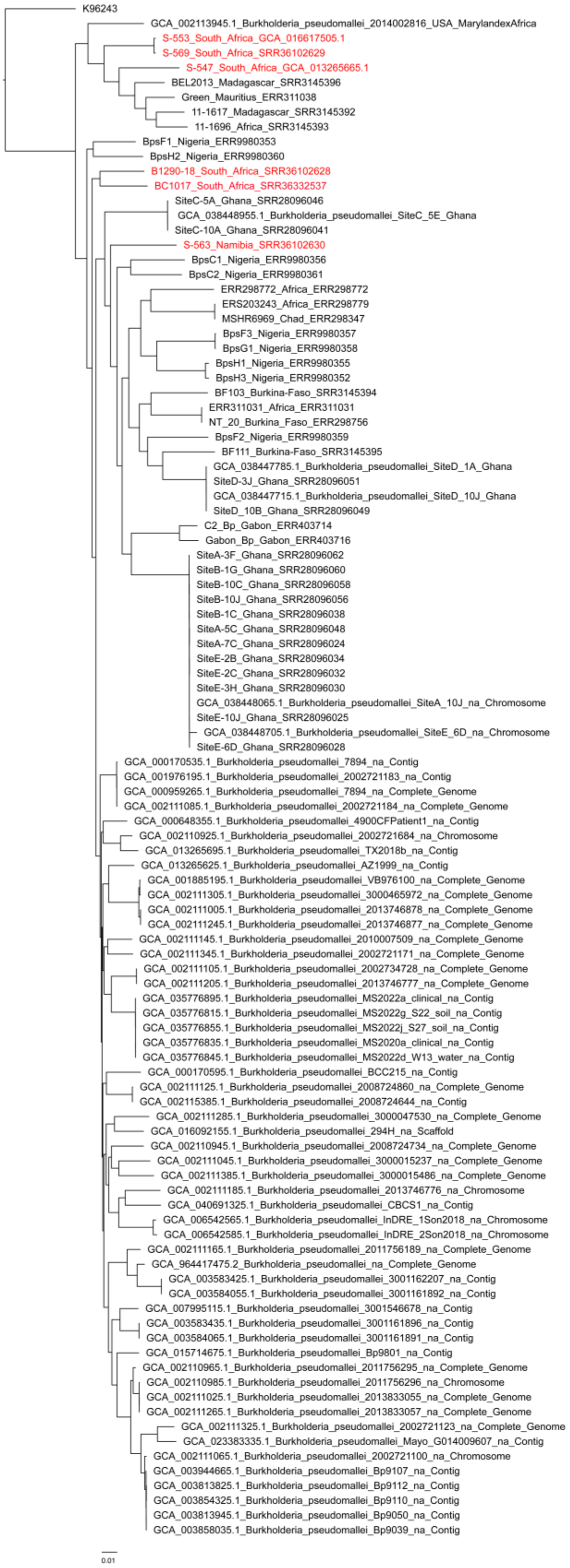

Figure S1. Maximum-likelihood phylogeny of core-genome SNPs from genomes within the Africa/America clade. Genomes sequenced in the present study are highlighted in red. While the America clade is collapsed in the main text for clarity, here the expanded topology is shown to display the detailed relationships among genomes from the Americas.
